# Antibody isotype diversity against SARS-CoV-2 is associated with differential serum neutralization capacities

**DOI:** 10.1038/s41598-021-84913-3

**Published:** 2021-03-10

**Authors:** Maria G. Noval, Maria E. Kaczmarek, Akiko Koide, Bruno A. Rodriguez-Rodriguez, Ping Louie, Takuya Tada, Takamitsu Hattori, Tatyana Panchenko, Larizbeth A. Romero, Kai Wen Teng, Andrew Bazley, Maren de Vries, Marie I. Samanovic, Jeffrey N. Weiser, Ioannis Aifantis, Joan Cangiarella, Mark J. Mulligan, Ludovic Desvignes, Meike Dittmann, Nathaniel R. Landau, Maria Aguero-Rosenfeld, Shohei Koide, Kenneth A. Stapleford

**Affiliations:** 1 Department of Microbiology, NYU Grossman School of Medicine, 430 East 29th Street, New York, NY 10016 USA; 2 Perlmutter Cancer Center, NYU Langone Health, New York, NY 10016 USA; 3 Department of Medicine, NYU Grossman School of Medicine, New York, NY 10016 USA; 4 Department of Pathology, NYU Grossman School of Medicine, New York, NY 10016 USA; 5 Department of Biochemistry and Pharmacology, NYU Grossman School of Medicine, 521 1st Avenue, New York, NY 10016 USA; 6 New York University Langone Vaccine Center and New York University Grossman School of Medicine, New York, NY 10016 USA; 7 Office of Science and Research, NYU Langone Health, New York, NY 10016 USA

**Keywords:** SARS-CoV-2, Antibodies

## Abstract

Understanding antibody responses to SARS-CoV-2 is indispensable for the development of containment measures to overcome the current COVID-19 pandemic. Recent studies showed that serum from convalescent patients can display variable neutralization capacities. Still, it remains unclear whether there are specific signatures that can be used to predict neutralization. Here, we performed a detailed analysis of sera from a cohort of 101 recovered healthcare workers and we addressed their SARS-CoV-2 antibody response by ELISA against SARS-CoV-2 Spike receptor binding domain and nucleoprotein. Both ELISA methods detected sustained levels of serum IgG against both antigens. Yet, the majority of individuals from our cohort generated antibodies with low neutralization capacity and only 6% showed high neutralizing titers against both authentic SARS-CoV-2 virus and the Spike pseudotyped virus. Interestingly, higher neutralizing sera correlate with detection of -IgG, IgM and IgA antibodies against both antigens, while individuals with positive IgG alone showed poor neutralization response. These results suggest that having a broader repertoire of antibodies may contribute to more potent SARS-CoV-2 neutralization. Altogether, our work provides a cross sectional snapshot of the SARS-CoV-2 neutralizing antibody response in recovered healthcare workers and provides preliminary evidence that possessing multiple antibody isotypes can play an important role in predicting SARS-CoV-2 neutralization.

## Introduction

The novel coronavirus, Severe Acute Respiratory Syndrome Coronavirus 2 (SARS-CoV-2), has rapidly spread across the globe, leading to Coronavirus Disease 2019 (COVID-19), devastating mortality, and significant impacts on our way of life. One question that still remains is whether those infected by SARS-CoV-2 generate an immune response that will protect them from reinfection^1^. Moreover, this question is particularly important for the development of a SARS-CoV-2 vaccine, as an effective vaccine would need to generate a potent neutralizing antibody response and immunological memory to provide long-lasting protection^2,3^. Thus, it is essential that we carefully study and document the neutralizing antibody responses in recovered individuals.

SARS-CoV-2 antibody testing is critical to understanding who has been infected and to provide a picture of seroprevalence in a community^4–9^. However, while these tests are important and provide a relative antibody titer, they are seen more as a “yes or no” type of answer to whether an individual has been infected. Importantly, these tests do not provide information on whether the SARS-CoV-2-specific antibodies present in serum are protective, including through virus neutralization, and as such, a positive antibody test may give individuals a false sense of “immunity” to the virus.

**Figure 1:**
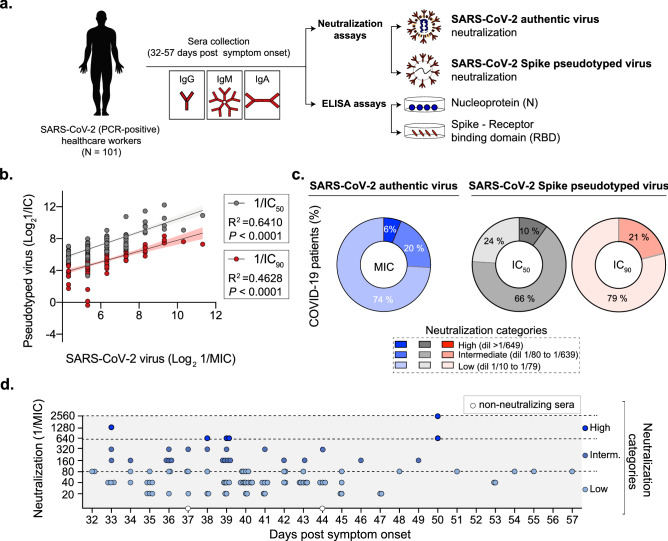
SARS-CoV-2 neutralizing antibody response. (**a**) Schematic representation of the experimental design. (**b**) Correlation analysis of the sera neutralization level of 101 COVID-19 convalescent patients. The data presented are the log2 of the neutralization titer against the authentic SARS-CoV-2 virus (1/MIC) and the Spike pseudotyped virus 1/IC_50_ or 1/IC_90_. Correlation and linear regression analyses were performed using GraphPad Prism 8. P values were calculated using a two-sided *F* test. (**c**) SARS-CoV-2 neutralization categories. Sera of convalescent patients was defined as low (dil 1/10 to 1/80), intermediate (dil 1/81 to 1/639) and high (dil > 1/640) based on authentic SARS-CoV-2 virus or Spike pseudotyped virus. (**d**) Distribution of authentic SARS-CoV-2 virus neutralization titers (1/MIC) over days post symptom onset. Dashed lines indicate the neutralization levels as defined in (**b**). White dots indicate two individuals with SARS-CoV-2 non-neutralizing sera.

A number of studies have begun to unravel the antibody response to SARS-CoV-2 beyond a simple “yes or no” answer^9–18^. Antibody responses to SARS-CoV-2 can have variable neutralization capacities^19–21^. Still, it remains unclear whether there are specific signatures that can be used to predict neutralization. Here, we hypothesized that by examining the antibody profile in patient’s serum in terms of antigens, antibody isotypes (IgG, IgM, and IgA), and neutralization, we would be able to identify specific signatures associated to effective SARS-CoV-2 neutralization. In this study, we obtained convalescent serum from 101 SARS-CoV-2 PCR-positive healthcare workers and performed a comprehensive analysis of neutralization of authentic SARS-CoV-2 and a Spike pseudotyped lentivirus as well as serum IgG, IgM, and IgA antibody titers to the SARS-CoV-2 spike receptor-binding domain (RBD) and the nucleoprotein (N) (Fig. 1a). Altogether, this study provides a cross sectional snapshot of the SARS-CoV-2 neutralizing antibody response in recovered healthcare workers and provides preliminary evidence of potential important signatures to predict SARS-CoV-2 seroneutralization capacities.

## Results

### SARS-CoV-2 seroneutralization capacity is low to intermediate in the majority of individuals

To better understand the human SARS-CoV-2 neutralizing antibody response in convalescent patients, we obtained serum from 101 COVID-19-recovered New York City healthcare workers who had experienced symptoms and had tested positive (by PCR testing) for SARS-CoV-2 in March 2020 (Fig. 1a and Supplementary Table 1). To begin, we assessed how well each individual’s serum was able to neutralize both authentic SARS-CoV-2 (isolate USA-WA01/2020), and a lentiviral pseudotyped virus bearing the SARS-CoV-2 Spike protein (pseudotyped virus) in vitro. Pseudotyped viruses are a safe alternative to authentic virus assays^22^, and can therefore be employed by a greater number of research institutes and clinical laboratories to assess neutralization of convalescent serum. However, several studies have shown differences in sensitivity between neutralization of authentic SARS-CoV-2 and specific pseudotyped viruses^23,24^. Thus, we set out to determine serum neutralization capacity using both our in-house lentiviral pseudotyped virus and the authentic SARS-CoV-2 USA-WA01/2020 isolate.

For the neutralization assays performed with authentic SARS-CoV-2 virus we determined the minimum inhibitory concentration (MIC), calculated as the minimum serum dilution at which serum is fully-protective (Supplementary Table 1). For the neutralization experiments performed with the Spike pseudotyped virus, we used a reporter pseudotyped virus expressing luciferase (Supplementary Fig. 1 and Supplementary Table 1) and we determined both the IC_50_ as well as the IC_90_. Using these systems, we found that neutralization of the authentic SARS-CoV-2 and the Spike pseudotyped virus was positively correlated (Fig. 1b, R^2^ = 0.64, p < 0.0001 for IC_50_ and R^2^ = 0.49, p < 0.0001 for IC_90_). In addition, we found that SARS-CoV-2 neutralizing antibody response was diverse in magnitude in our cohort with samples exhibiting high, medium and low neutralization capacities for both authentic as well as Spike pseudotyped viruses (Fig. 1c). The majority COVID-19 patients from our cohort had serum with low to intermediate neutralization capacity (dilution 1/10 to 1/640) and only a select few (~ 6 to 10%) displayed high neutralization (dilution > 1/640) against both authentic and Spike pseudotyped virus (Fig. 1c).

Finally, because samples were collected from individuals at varying times post onset of symptoms, we asked whether the sample collection time points contributed to the observed differences in the sera neutralization capacities. We found that there was no particular pattern in this cohort, with individuals displaying low, medium, and high neutralizing sera against authentic virus found between 32 and 57 days (Fig. 1d). Together, these results show that at the time points we analyzed (32–57 days post symptom onset), serum SARS-CoV-2 neutralizing antibody capacity is low to intermediate in most recovered individuals.

### Human SARS-CoV-2 infection generates antigen-specific, multi-isotype antibody response

Given the broad neutralization capacity observed in this cohort, we were interested in examining the antibody profile of each individual. It is not uncommon that antibody responses are skewed toward one or a few viral proteins^10,25^. If this is the case, using a single antigen may result in a biased test that inadequately detects antibodies produced by convalescent individuals. Thus, we quantified serum antibody IgG, IgA, and IgM titers by ELISA, focusing on antibodies generated to two SARS-CoV-2 antigens (Supplementary Table 1). First, we used the Gold Standard ELISA assay currently deployed at the Tisch Hospital Clinical Labs in New York City which uses the nucleoprotein (N). Second, we developed an in-house ELISA for the SARS-CoV-2 Spike receptor-binding domain (RBD) (Supplementary Fig. 2). If antibody titers against Spike RBD and N correlate, this suggests that individuals mount a uniform response to both these antigens. A lack of correlation suggests that individuals mount a skewed response toward one of these antigens preferentially.

**Figure 2:**
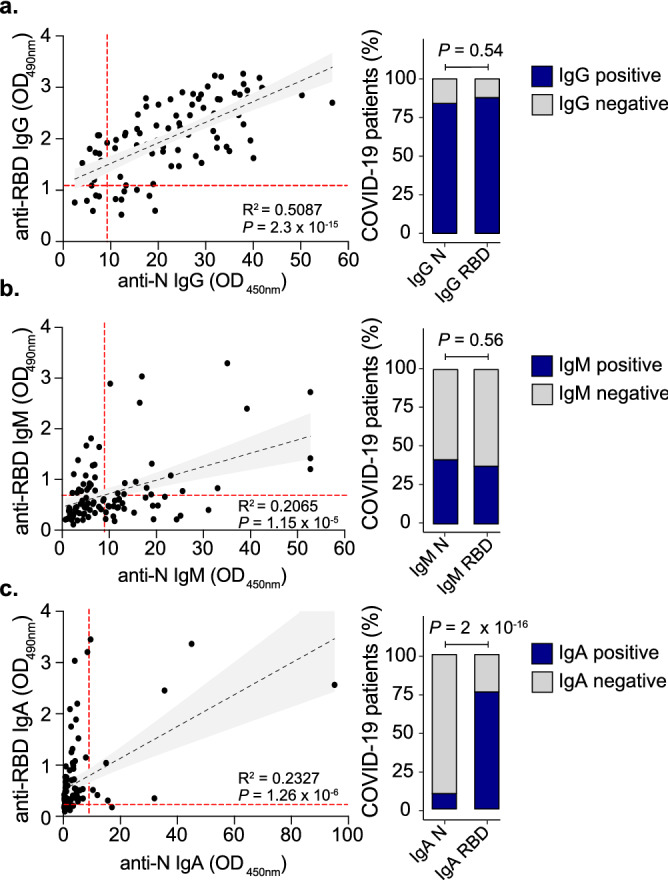
Isotype composition of SARS-CoV2 convalescent serum. Anti-RBD and anti-N ELISA correlation for IgG (**a**), IgM (**b**) and IgA (**c**). Left panels: correlations and linear regressions comparing anti-RBD and anti-N for each antibody isotype (N = 101). Analyses were performed using GraphPad Prism 8. P values were calculated using a two-sided *F* test. The red dashed lines indicate the threshold (anti-N ELISA for -IgG, -IgM or -IgA are OD450nm = 9; anti-RBD ELISA for -IgG is OD490nm = 1.091; -IgA is OD490nm = 0.256 and -IgM is OD490nm = 0.694) for each ELISA. Right panels indicate the percentage of COVID-19 PCR-positive patients that are positive (blue bars) or negative (gray bars) for IgG (**a**), IgM (**b**) or IgA (**c**). P values were calculated using two-sided Fisher’s exact test.

When we compared the isotype responses to anti-RBD and anti-N directly, we found that IgG correlated the strongest (R^2^ = 0.51) followed by IgA (R^2^ = 0.23) and IgM (R^2^ = 0.21) (Fig. 2a–c, left panels). The percentage of IgG and IgM positive individuals, as detected by reactivity against RBD or N, was similar (Fig. 2a,b, right panels). Strikingly, the majority of individuals with positive titers of IgA to the Spike RBD were largely IgA-negative for N (Fig. 2c, left panel). This translated to a significant difference in IgA detection between the Gold Standard test and our in-house ELISA (Fig. 2c, right panel, p = 2 × 10^–16^), and suggests that the IgA response is skewed towards the Spike RBD in comparison to N (Fig. 2c). It is possible that days post symptom onset plays a role in both serum antibody titers and the relative percentage of IgG, IgM and IgA positive people. Within our cohort we observed no relationship between time post symptom onset and antigen-specific antibody isotype titers (Supplementary Fig. 3), even finding IgM present at 50 days post infection (Supplementary Fig. 3b). Together these results suggest that the antibody isotype response to SARS-CoV-2 may be antigen-specific, with IgA skewed towards the RBD.

### SARS-CoV-2 neutralization best correlates with RBD antibody response

**Figure 3:**
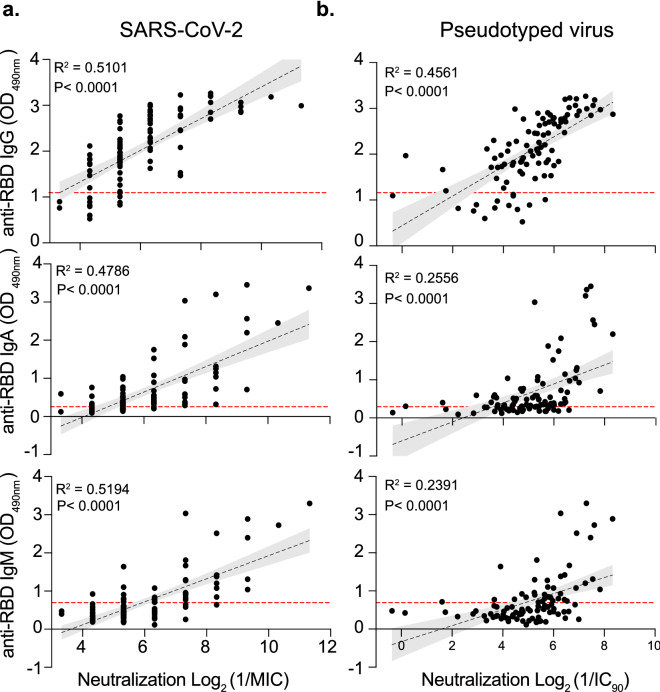
Correlation of anti-RBD antibody isotypes with viral neutralization. Anti-RBD ELISA correlation for IgG (top), IgA (middle) and IgM (bottom) with viral neutralization using authentic SARS-CoV-2 (**a**) or Pseudotyped virus (**b**). Correlation and linear regression analyses were performed using GraphPad Prism 8. P values were calculated using a two-sided *F* test. The red dashed lines indicate the threshold (anti-RBD ELISA for -IgG is OD490nm = 1.091; -IgA is OD490nm = 0.256 and -IgM is OD490nm = 0.694) for each ELISA.

To better understand how each antigen-specific antibody isotype correlated with neutralization, we compared the ELISA assay antibody titers with virus neutralization. While all anti-RBD isotype titers correlated similarly with the neutralization level of the authentic SARS-CoV-2 (R^2^ = 0.4786 – 0.5194) (Fig. 3a), we found that Spike pseudotyped virus neutralization correlated best with IgG titers (R^2^ = 0.4561) compared with IgA (R^2^ = 0.2556) or IgM (R^2^ = 0.2391) (Fig. 3b). Interestingly, with the exception of anti-N IgG antibodies for the authentic virus (R^2^ = 0.5277; R^2^ = 0.255), other anti-N isotype titers showed weaker correlations for the authentic SARS-CoV-2 virus (Supplementary Fig. 4), suggesting that ELISA methods based on the Spike RBD may benefit from detection of additional isotypes, rendering them better suited as predictors of sera neutralization.

### Robust SARS-CoV-2 neutralization associates with high-titer, multi-isotype antibody responses

**Figure 4:**
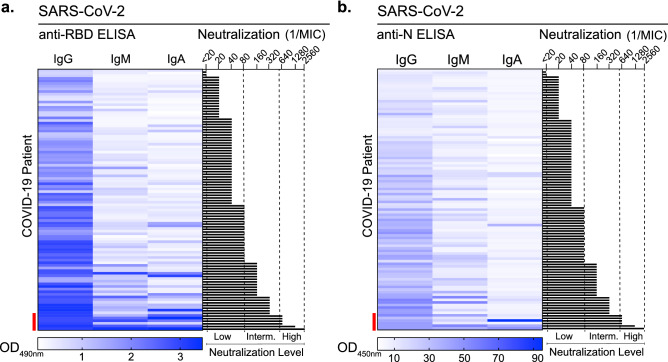
Identification of serological signatures for neutralization. Heatmap of anti-RBD (**a**) and anti-N (**b**) antibody isotype ELISA titers and corresponding authentic SARS-CoV-2 neutralization. Serological data from the 101 COVID-19 patients was ranked from low to high neutralization. Sera of convalescent patients was defined as low (dil 1/10 to 1/80), intermediate (dil 1/81 to 1/639) and high (dil > 1/640). Red bar indicates those COVID-19 patients with high neutralizer antibodies.

Next, we delineated what separates our highest neutralizers from the remainder of the cohort using the data collected on antigen-specific antibody isotype titers. We hypothesized that by examining the antibody profile in patient’s serum in terms of antigens, antibody isotypes (IgG, IgM, and IgA), and neutralization, we would be able to identify specific signatures associated to effective SARS-CoV-2 neutralization. To do so, we employed a holistic approach and compared the IgG, IgM and IgA titers for both anti-Spike RBD and anti-N antibodies against neutralizations level (Fig. 4a,b). This revealed that most of the high neutralizer sera (MIC higher or equal to 1/640) had high titers of IgG, IgM and IgA raised against both Spike RBD and N (Fig. 4a,b, red bar). The spike protein is the major determinant for virus neutralization and resides on the outside of the viral particle, exposed to the immune recognition. Consequently, we found stronger correlations between Spike-RBD isotypes compared with N isotypes, when comparing each individual’s IgG, IgM or IgA (anti-Spike RBD and N) against each other (Supplementary Fig. 5). However, in most cases the high neutralizers (red circles) had the highest antibody titers for all isotypes (Supplementary Fig. 5). These data suggest that mounting a robust antibody response, consisting of diverse isotypes, can contribute to efficient neutralization.

### Recovered individuals have multiple combinations of anti-SARS-CoV-2 serum antibodies

**Figure 5:**
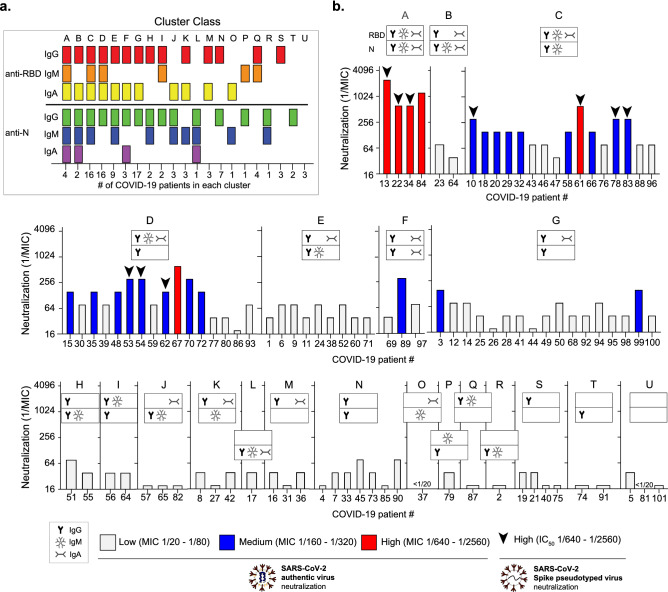
Clustering analysis of individual SARS-CoV-2 antibody response. (**a**) Combination of antibody isotypes in individual patient sera defining different cluster classes. ELISA titers were categorized as positive (ELISA titers > cutoff) or negative (ELISA titer < cutoff) for each sample for each individual antibody isotype. Clusters were made based on the presence or absence of specific isotypes (Cluster A to U). The number of patients in each cluster is shown on the x axis. (**b**) Neutralization levels shown for each individual antibody cluster.

Finally, given that we observed that having multiple isotypes associate with higher neutralization, we were interested in understanding the anti-RBD and N antibody signatures present in individuals and how they correlated with neutralization. Therefore, we classified whether the presence or absence of certain antigen-specific antibody isotypes (based on ELISA cutoffs) related to neutralization level. The 101 COVID-19 patients comprised 21 different antibody combinations, or “clusters” (Fig. 5a), ranging from being positive for all six antibodies (N/RBD IgG, IgA, IgM) to three individuals who did not have antibodies to neither N or RBD (Fig. 5a,b, Cluster U). We found that all the individuals with high neutralizing titers against authentic virus and/or Spike pseudotyped virus (dilution > 1/640) had sera tested positive for all the three isotypes (IgG, IgM and IgA) against Spike RBD (Fig. 5b, cluster A, C and D). Interestingly, 4 out of the 6 sera with high neutralizing titers (MIC = 1/640–1/2560) tested positive for all three isotypes (IgA, IgG, and IgM) responsive against both Spike RBD and N (Fig. 5b, Cluster A).

Notably, two individuals with high neutralization titers by authentic virus neutralization display intermediate neutralization when evaluated against Spike pseudotyped virus (sample # 84, cluster A and sample # 67, cluster D). Given that the spike pseudotyped virus only expressed SARS-CoV-2 spike protein, these differences might suggest the potential contribution of antibodies targeting other antigens in neutralization against authentic virus. Interestingly, Cluster B, which only lacks IgM against the RBD does not have high neutralization compared with Clusters A, C, and D, suggesting that specific antibody combinations and/or titers may contribute to maximum neutralization. However, other factors such as a more mature, efficient antibody response and levels of affinity can be contributing to this difference. Taken together, these results suggest that having IgG alone may not be enough for efficient neutralization, while having multiple isotypes at high titers present in serum may contribute to more potent SARS-CoV-2 neutralization.

## Discussion

Understanding the antibody response and potential immunity to SARS-CoV-2 is critical for global public health and the development of efficacious vaccines^1,3,26–28^. In this study, we performed a comprehensive analysis of the SARS-CoV-2-specific antibody response in 101 convalescent healthcare workers serum samples. We determined the neutralization capacity of the sera using both authentic virus and pseudotyped particles, quantified the titers of three antibody isotypes (IgG, IgM, and IgA) to both the spike RBD and the nucleoprotein, and investigated the correlation of neutralization and antibody levels. We found, that the serum SARS-CoV-2 neutralizing antibody capacity was low for the majority of recovered individuals. In extreme cases, we detected no antibodies against SARS-CoV-2 Spike RBD or N in three individuals. These observations are in line with previously published studies and may suggest that those infected do not produce efficient neutralizing antibodies or neutralizing antibodies rapidly wane by the time samples are collected^16,19,29,30^. Detailed longitudinal studies are beginning to emerge, showing that serum antibody levels are relatively stable or decrease over time^20,31,32^. In-line with our results these studies find a positive correlation between IgG titers and neutralization^31,32^. Further characterization of neutralization at early and late timepoints is necessary to correlate antibody titer stability to viral protection. However, it is essential to keep in mind that, while neutralization titers can be quantified by reliable laboratory assays, we do not know the overall protective capacity of antibodies against SARS-CoV-2 in patients. Thus, those with low neutralizing capacities in the lab (MIC < 1/80) could be efficiently protected from SARS-CoV-2 reinfection. Detailed studies monitoring initial and possible reinfections along with the antibody response are crucial to understand immunity to SARS-CoV-2.

To further understand the composition of convalescent sera, we quantified the amounts of IgG, IgA, and IgM targeting the spike protein RBD and N. We found 21 different antibody combinations that did not directly correlate with days post symptom onset, yet did associate with antibody neutralization. Of these combinations, the majority of individuals fell into four distinct clusters comprised of different antigen-specific isotypes. It remains to be elucidated how these clusters are generated and why certain clusters elicit potent SARS-CoV-2 neutralization (Cluster A) and others do not (Cluster B). Since we use a targeted approach focusing on only two antigens and specific epitopes, it is possible that there are other anti-SARS-CoV-2 antibodies that bind viral particles and impact neutralization^33^.

Longitudinal analysis of COVID-19 convalescent patients, have shown that serum IgM antibodies can persist for up to 80 days post symptom onset^34^. In line with this observation we found high quantities of IgM present in some individuals from 32 to 50 days post symptom onset. These findings are intriguing since IgM antibodies are usually considered as a marker of a recent infection and their circulating titers are thought to decrease as class switching occurs to IgG and IgA. In this situation, looking at multiple isotypes including IgA as well as multiple antigens may be beneficial in determining the relative infection timeline. Along these lines, we also found that individuals having multiple antibodies isotypes to both RBD and N had the best neutralization against both authentic and Spike pseudotyped virus. Moreover, we found that individuals who had IgG in combination with IgM and IgA against the RBD had the greatest neutralization capacity, suggesting that having a broader repertoire of antibodies may contribute to more potent SARS-CoV-2 neutralization. However, other factors such as, age, maturity of the response, severity of disease, might contribute as well. It has been shown that severity of disease can play an important role in the antibody response and it may be that in these individuals, disease is a driver for multiple isotypes and increased neutralization^15,35^. Thus, understanding how enhanced disease burden leads to the generation of multiple neutralizing isotypes will be crucial for vaccine development.

These results, particularly the presence of individuals with low neutralizing antibodies are interesting as each individual in our cohort has recovered from SARS-CoV-2 infection. This observation suggests that either we missed the period of robust neutralizing antibody production in certain individuals or other mechanisms, such as potent SARS-CoV-2 specific T-cell responses^36–42^, provided effective viral clearance and immunity. Future studies coupling assessment of antibody and T-cell responses with key clinical information (age, sex, severity of symptoms) will be essential to fully understand this complex immune response. Moreover, these results also highlight the need to study the SARS-CoV-2 genetics as the virus encodes multiple undefined accessory proteins that could impact the immune response during infection. Additionally, longitudinal studies are necessary to fully understand the complex immune response to SARS-CoV-2 in the hopes to contain this virus and to prepare for the emergence of related viruses.

## Methods

### Serum samples

Serum was collected from 101 healthcare workers from NYU Langone who had laboratory evidence of COVID-19 (PCR-positive) ranging from 32 to 57 days post symptoms onset. Healthy control sera were collected from patients through the NYU Vaccine Center. All methods were performed in accordance with the guidelines and regulations of the New York University Institution Review Board. The study and experimental protocols were approved by the New York University Institutional Review Board. All patients gave written informed consent and all samples were deidentified for this study under IRB #i20-00595 (SARS-CoV-2 infected) and IRB #s18-02037 (healthy controls).

### Plasmids

Spike pseudotyped virus generation: to construct the SARS-CoV-2 SΔ19 expression vector pcCOV2-Δ19.S, a codon-optimized DNA sequence was synthesized encoding the SARS-CoV-2 Spike (Wuhan-Hu-1/2019). An amplicon was amplified encoding the S protein with a 19 amino acid truncation of the cytoplasmic tail using primers containing flanking 5′-KpnI and 3′-XhoI sites and cloned into pcDNA6 (Invitrogen, Inc.). To construct the ACE2 expression vector pLenti.ACE2-HA, the ACE2 coding sequence was amplified from an ACE2 cDNA (Origene Inc) using primers with flanking 5′-XbaI and 3′-SalI sites and cloned into pLenti.CMV.GFP.puro. For RBD ELISA: The nucleotide sequence of the RBD of SARS-CoV-2 isolate Wuhan-Hu-1 (residues 328–531) was obtained from a GenBank entry MN908947.3. A codon-optimized gene encoding the RBD with a hexa-histidine tag (His_6_-tag) and biotinylation tag (Avi-tag) at the C terminus was synthesized (Integrated DNA Technologies) and cloned into the pBCAG mammalian expression vector.

### Cells and virus

Vero E6 cells (ATCC CRL-1586) were maintained in Dulbecco’s Modified Eagle’s Medium (DMEM, Corning) supplemented with 10% fetal bovine serum (FBS, Atlanta Biologics) and 1% nonessential amino acids (NEAA, Corning). Human embryonic kidney (HEK) 293T cells were maintained in DMEM supplemented with 10% FBS, and 1% penicillin/streptomycin (P/S). To generate 293T stably expressing ACE2 (ACE2-293T cells), 293T cells were transfected with pLenti.ACE2-HA DNA by lipofection with lipofectamine 2000 (Invitrogen). After 2 days, the cells were selected in DMEM supplemented with 10% FBS, 1% P/S, and 1 μg/ml of puromycin. Single cell clones were expanded and analyzed by flow cytometry for ACE2 expression and a single clone was chosen for subsequent use. Expi293T cells (Thermo Fisher) were maintained in Expi293 Expression Medium (Thermo Fisher). All cells were maintained at 37 °C with 5% CO_2_ and confirmed mycoplasma free.

SARS-CoV-2, isolate USA-WA1/2020^43^ (BEI resources # NR52281, a gift from Dr. Mark Mulligan at the NYU Langone Vaccine Center) was amplified once in Vero E6 cells (P1 from the original BEI stock). Briefly, 90–95% confluent T175 flask (Thomas Scientific) of Vero E6 (1 × 10^7^ cells) was infected with 10 μl of the BEI stock in 3 ml of infection media (DMEM, 2% FBS, 1% NEAA, and 10 mM HEPES, pH 7.0) for 1 h. After 1 h, 15 ml of infection media was added to the inoculum and cells were incubated 72 h at 37 °C and 5% CO_2_. After 72 h, the supernatant was collected and the monolayer was frozen and thawed once. Both supernatant and cellular fractions were combined, centrifuged for 5 min at 1200 rpm, and filtered using a 0.22 μm Steriflip (Millipore). Viral titers were determined by plaque assay in Vero E6 cells. In brief, 220,000 Vero E6 cells/well were seeded in a 24 well plate, 24 h before infection. Ten-fold dilutions of the virus in DMEM were added to the Vero E6 monolayers for 1 h at 37 °C. Following incubation, cells were overlaid with 0.8% agarose in DMEM containing 2% FBS and incubated at 37 °C for 72 h. The cells were fixed with 10% formalin, the agarose plug removed, and plaques visualized by crystal violet staining. All experiments with authentic SARS-CoV-2 were conducted in the NYU Grossman School of Medicine ABSL3 facility.

### Spike pseudotyped virus preparation

Spike pseudotyped virus was produced by calcium phosphate co-transfection of 293T cells with pMDL, plenti.GFP-NLuc, pcSARS-CoV-2-SΔ19 and pcRev at a ratio of 4:3:4:1. The supernatant was harvested 2 days post-transfection, passed through an 0.22 μm filter and then pelleted by ultracentrifugation for 90 min at 30,000 rpm in an SW40.1 rotor. The pellet was resuspended in 1/10th the original volume of DMEM supplemented with 10% FBS and frozen at − 80 °C in aliquots.

### SARS-CoV-2 neutralization assay

Vero E6 cells (30,400 cells/well) were seeded in a 96 well plate 24 h before infection so that a monolayer was present the following day. Serum samples from COVID-19 convalescent individuals and healthy donors were two-fold serially diluted (spanning from 1:10 to 1:10,240) in DMEM (Corning), 1% NEAA (Corning) and 10 mM HEPES (Gibco). Diluted serum samples were mixed 1:1 (vol/vol) with SARS-CoV-2 virus (6.8 × 10^3^ PFU/ml), and incubated 1 h at 37 °C. During the incubation period, Vero E6 monolayers were washed once with DMEM (Corning) to remove any serum present in the media that could interfere with the assay. After incubation, 100 μl of the serum:SARS-CoV-2 mixtures were added to the Vero E6 monolayers, and cells were incubated at 37 °C. Cells were monitored every day for cytopathic effects (CPE) induced by viral infection, and at 5 days post infection cells were fixed in 10% formalin solution (Fisher Scientific) for 1 h. Cells were then stained by adding 50 µl crystal violet/well and incubated for 30 min. Each well was scored as “0” if there was no monolayer left, “0.5” if there was some monolayer and the well was clearly infected, or “1” if the monolayer was intact. The minimum inhibitory concentration (MIC) was defined as the minimal serum dilution in which the cell monolayer was intact (score = 1). Each serum sample was measured in technical duplicates.

### Spike pseudotyped virus neutralization assay

To determine neutralizing serum titers, ACE2-293T cells were plated in 96 well tissue culture dishes at 10,000 cells/well. The following day, twofold dilutions of the donor sera were made in culture medium spanning a range from 1:10 to 1:10,240. Each dilution (50 μl) was mixed with 5 μl SARS-CoV-2 SΔ19 lentiviral pseudotype. The mixtures were incubated for 30 min at room temperature and then added to the plated ACE-2 293T cells. The plates were cultured for two days at 37 °C with 5% CO_2_ after which the supernatant was removed and replaced with 50 μl Nano-Glo Luciferase Substrate (Promega, Inc.). Light emission was measured in an Envision 2103 Multi-label plate reader (PerkinElmer, Inc.).

### SARS-CoV-2 nucleoprotein ELISA

Serum IgG, IgA, and IgM antibodies to the SARS-CoV-2 nucleoprotein were tested using the SARS-CoV-2 IgG, IgA and IgM ELISA kits manufactured by Virotech Diagnostics GmbH for Gold Standard Diagnostics (Davis, CA) following the manufacturer’s instructions. For the detection of IgG and IgA, serum samples were diluted 1:100 in dilution buffer and for IgM, serum samples were diluted 1:101 in RF-Adsorption dilution buffer mixture and incubated at room temperature for 15 min before being added to the wells. Results are reported qualitatively as negative (< 9.0 units), equivocal (9.0–11.0 units), and positive (> 11.0 units).

### SARS-CoV-2 spike receptor-binding domain (RBD) purification and ELISA

The Expi293F cells (Thermo Fisher) were transiently transfected with the expression vector using the ExpiFectamine 293 Transfection Kit (Thermo Fisher, A14524)) and the Expi293 Expression Medium (Thermo Fisher, A14351). The transfected cells were cultured at 37 °C with 8% CO_2_ for 7 days. The culture supernatant was harvested by centrifugation, supplemented with protease inhibitors and clarified by further centrifugation at 8000 rpm for 20 min and filtration through a 0.22 µm filter. The supernatants were dialyzed into 20 mM sodium phosphate pH 7.4 with 500 mM sodium chloride and the recombinant RBD was purified by immobilized metal ion affinity chromatography (IMAC) using a HisTrap excel column (GE Healthcare). The purified protein was biotinylated using the *E. coli* BirA enzyme produced in house in the presence of 10 mM ATP and 0.5 mM biotin. The RBD protein was purified by IMAC and dialyzed against PBS and stored at − 80 °C. High purity of the purified protein was confirmed using SDS-PAGE, and analysis by size exclusion chromatography using a Superdex 75 10/300 Increase column (GE Healthcare), which showed a single, monodisperse peak consistent with its molecular mass (Supplementary Fig. 2a and b).

For the ELISA, the wells of 384-well ELISA plates (NUNC Maxisort cat# 464718) were coated with 15 µl of 4 µg/ml neutravidin (Thermo Fisher cat# 31000) for 1 h at room temperature (R.T.) in a humidified chamber. The wells were washed with 100 µl PBST (PBS containing 0.1% Tween 20) (Thermo Fisher, cat# BP337-500) three times using a BioTek 405TS plate washer housed in a BSL-2 biosafety cabinet. The wells were blocked with 0.5% BSA (Gemini Bio cat# 700-100P, skim milk was not used for blocking because milk can contain biotin that would inhibit antigen immobilization) in PBS overnight at 4 °C. After removing the blocking buffer, 15 µl of 20 nM RBD-His_6_-Avi-biotin in PBS was added to each well using a Mantis dispenser (Formulatrix). The plates were incubated at R.T. in a humidified chamber for 20 min, and the wells were washed with 100 µl PBST three times using the plate washer. The wells were further blocked with 15 µl of 10 µM biotin in 3% skim milk in PBST (Sigma, cat# 1.15363.0500) for ten min at R.T. in order to block unsaturated neutravidin.

Serum samples were heat-treated at 56 °C for 1 h^9^ and diluted 158-fold in 1% skim milk in PBST and placed in a 96-well polypropylene plate (Thermo Fisher, cat # AB-0796), which served as a master plate. The following sample handling was performed using a Hudson SOLO liquid handler housed in a BSL-2 biosafety cabinet. Twelve microliters of diluted serum were transferred per well of an RBD-immobilized 384-well plate after removing the biotin solution. To prepare 500-fold diluted samples, 8.22 µl of the diluent (1% skim milk in PBST) was first dispensed into a well to which 3.78 µl of diluted serum from the master plate was added. After 2 h of incubation at R.T., the plate was washed with PBST three times using the plate washer. 15 μl of secondary antibody (anti human Fab-HRP: Jackson ImmunoResearch, cat# 109-035-097; anti human IgG-HRP: Sigma, cat# A6029-1ML; anti human IgM-HRP: Jackson ImmunoResearch, cat# 109-035-129; and anti-human IgA-HRP: Jackson ImmunoResearch, cat# 109-035-011; × 1/5000 diluted in 1% milk in PBST) was added using the Mantis dispenser and incubated for 1 h at R.T. After washing the plate with PBST three times and then with PBS three times, 25 µl of the substrate (SIGMAFAST OPD; Sigma P9187-50SET) was added into the wells using the Mantis dispenser. Subsequently, the stop solution (25 µl of 2 M HCl) was added using the Mantis dispenser. The dispenser was programmed in such a way that the reaction in each well was stopped after 10 min. Absorbance at 490 nm was measured using a BioTek Epoch 2 plate reader. All samples were analyzed two independent times to identify outliers. Those yielding inconsistent results in both assays were excluded from analysis (Sample 76 and 86, Supplementary Table 1). Thresholds were determined comparing the 101 SARS-CoV-2 PCR positive samples at two dilutions 1/158 and 1/500 and compared with 43 SARS-CoV-2 PCR negative individuals. The cutoff values were defined as the mean plus three times the standard deviation (SD) of the negative control samples. Results were reported as positive if the values are > 1.091 for IgG, > 0.256 for IgA and > 0.694 for IgM (Supplementary Fig. 2c).

### Data analysis and statistics

All experiments were performed in technical duplicates and data analysis and statistics were performed using GraphPad Prism (Version 8.4.3), R Studio (Version 1.2.5001), and R (Version 3.6.3).

## Supplementary information

Supplementary information.

## Data Availability

Data that supports all figures, supplementary data, and analysis are found in this article, Supplementary Table 1 and Supplemental Data.
