# Supplementary material for: Antibody isotype diversity against SARS-CoV-2 is associated with differential serum neutralization capacities
Source: Sci Rep. 2021 Mar 10;11:5538. doi: 10.1038/s41598-021-84913-3 (PMC7946906; doi:10.1038/s41598-021-84913-3)
Supplement: Supplementary file 1 — Supplementary information. [file 41598_2021_84913_MOESM1_ESM.pdf]

Antibody isotype diversity against SARS-CoV-2 is associated with differential serum neutralization capacities.

Maria G. Noval[1†], Maria E. Kaczmarek[1†], Akiko Koide[2,3†], Bruno A. Rodriguez-Rodriguez[1†], Ping Louie[4†], Takuya Tada[1†], Takamitsu Hattori[2,5], Tatyana Panchenko[2], Larizbeth A. Romero[5], Kai Wen Teng[2], Andrew Bazley[5], Maren de Vries[1], Marie I. Samanovic[6], Jeffrey N. Weiser[1], Ioannis Aifantis[2,4], Joan Cangiarella[4], Mark J. Mulligan[6], Ludovic Desvignes[3,6,7], Meike Dittmann[1], Nathaniel R. Landau[1], Maria Aguero-Rosenfeld[4], Shohei Koide[2,5*], and Kenneth A. Stapleford[1*]

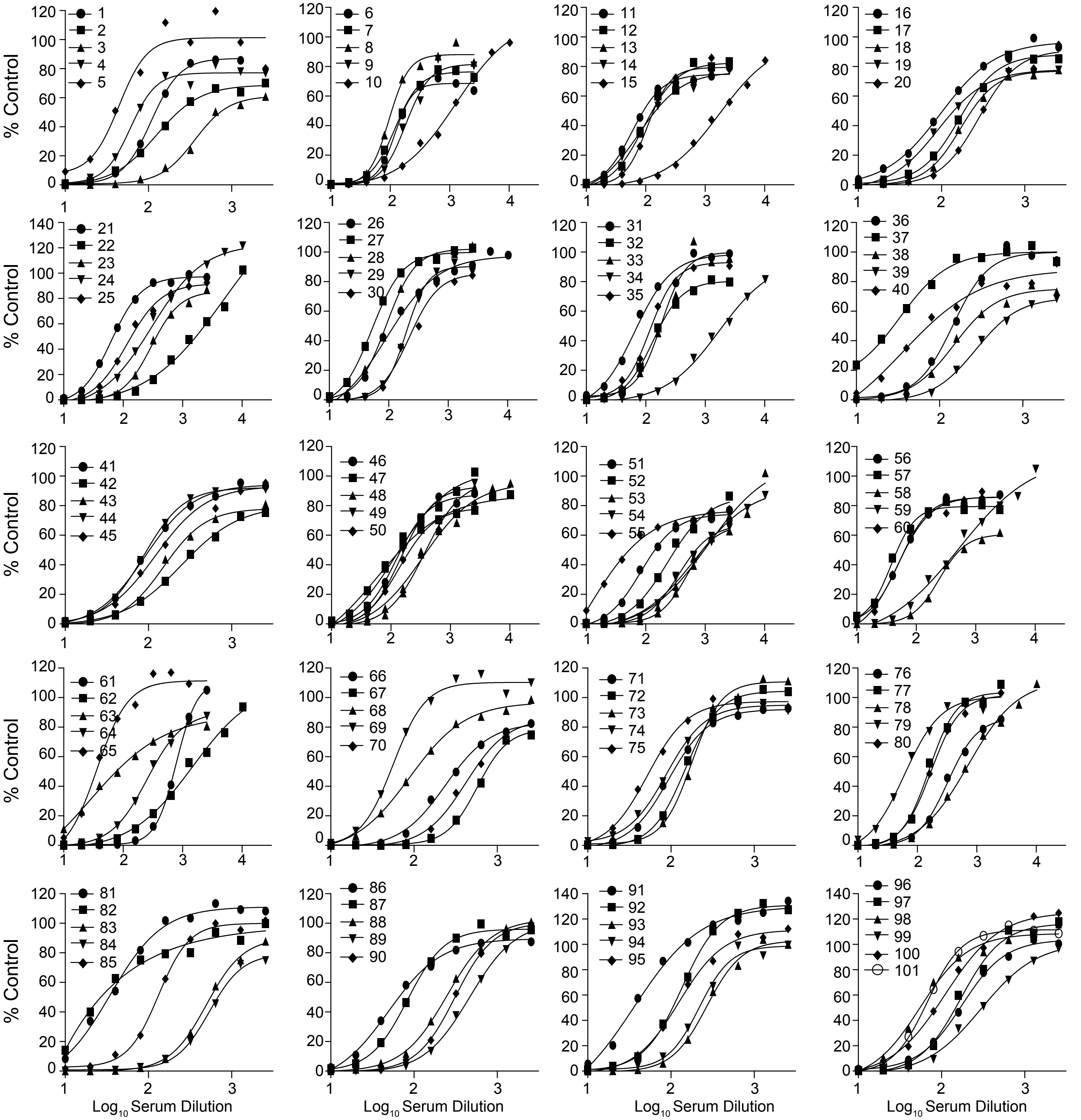


**Supplementary Figure 1. Individual sera neutralization of pseudotyped virus.** Pseudotyped virus was mixed 1:1 with 2-fold dilutions of individual sera and incubated at room temperature for 30 minutes before infecting ACE2-293T cells. Relative infection was determined by luciferase expression 48 hrs post-incubation. Data is represented as the percentage of the untreated control. Data was fitted to a variable slope model log(serum dilution) versus response using Graph Pad prism.

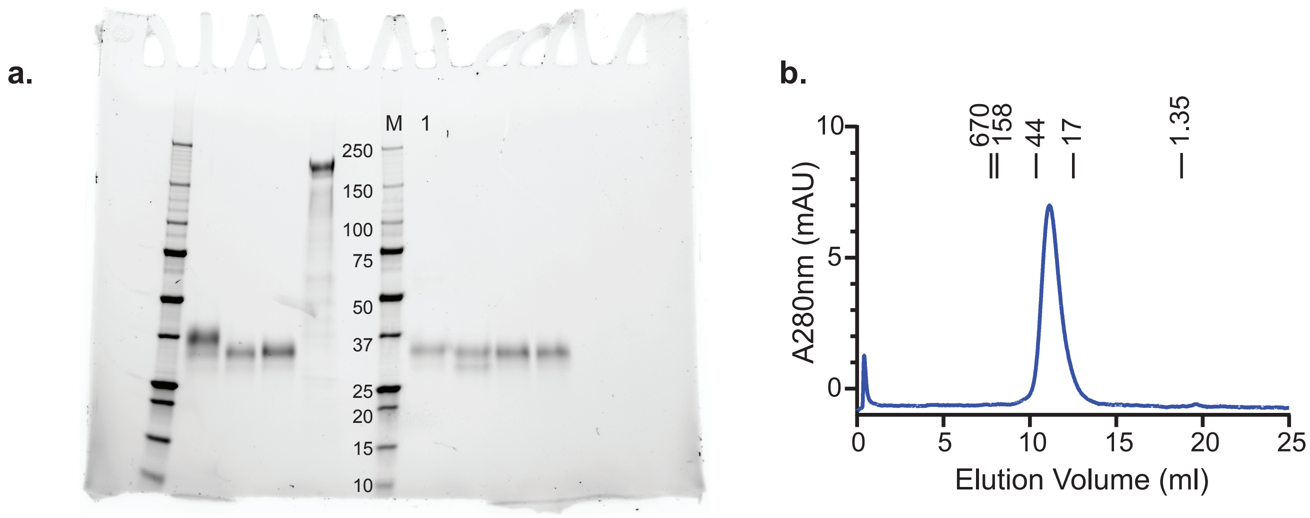


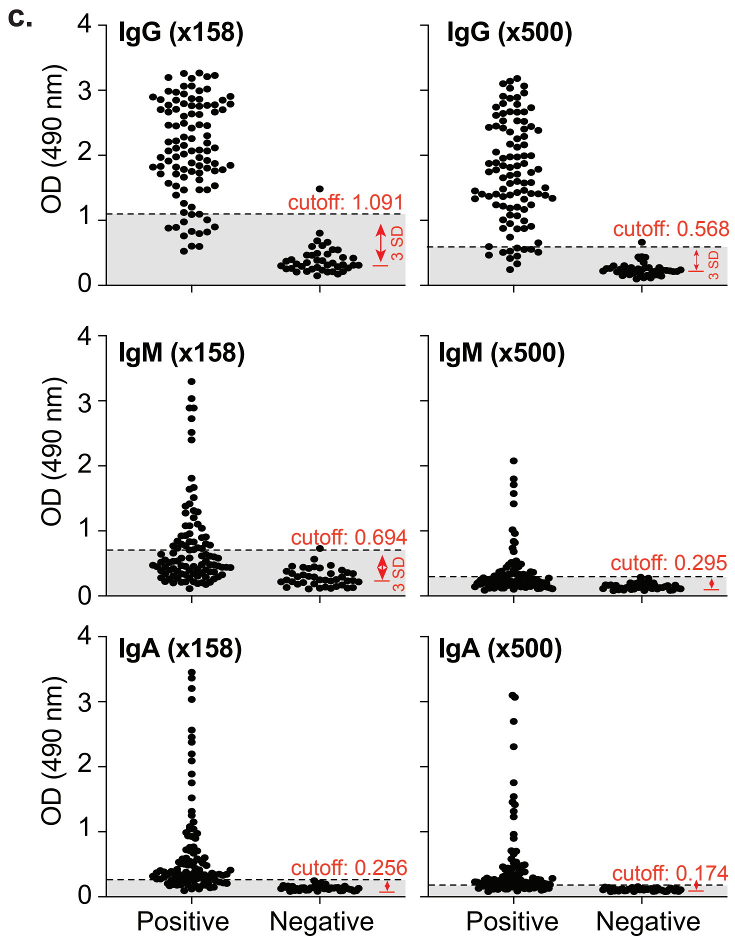


**d.**

| Isotype | Serum dilution | Sensitivity | Specificity |
|---|---|---|---|
| IgG | 158 | 0.881 | 0.976 |
| IgG | 500 | 0.901 | 0.976 |
| IgM | 158 | 0.386 | 0.976 |
| IgM | 500 | 0.386 | 1.000 |
| IgA | 158 | 0.762 | 1.000 |
| IgA | 500 | 0.752 | 1.000 |

**Supplementary Figure. 2. Production of SARS-CoV-2 Spike RBD antigen and characterization of anti-RBD ELISA. a.** SDS-PAGE of purified RBD-His6-Avi-biotin (lane 1) using the Bio-Rad stain-free detection method. Lane M contains standards with its molecular weight in kDa. The other lanes contain unrelated samples. **b.** Purified RBD-His6-Avi-biotin size-exclusion chromatography on a Superdex 75 10/300 Increase column detected using absorbance at 280 nm. The elution positions of molecular weight standards are marked as bars with their molecular weights in kDa. **c.** Determination of ELISA thresholds. Serum from the 101 SARS-CoV-2 PCR positive were diluted 1/158 and 1/500 and compared with 43 SARS-CoV-2 PCR negative individuals. The cutoff values were defined as the mean plus three times the standard deviation (SD) of the negative control samples as shown by the red arrow. The dashed line indicates the position of each individual cutoff. Cutoff values are indicated in red. **d.** Table showing the sensitivity (% of true positive in positive) and specificity (% of true negative in negative) of each RBD antibody subtype and dilution.

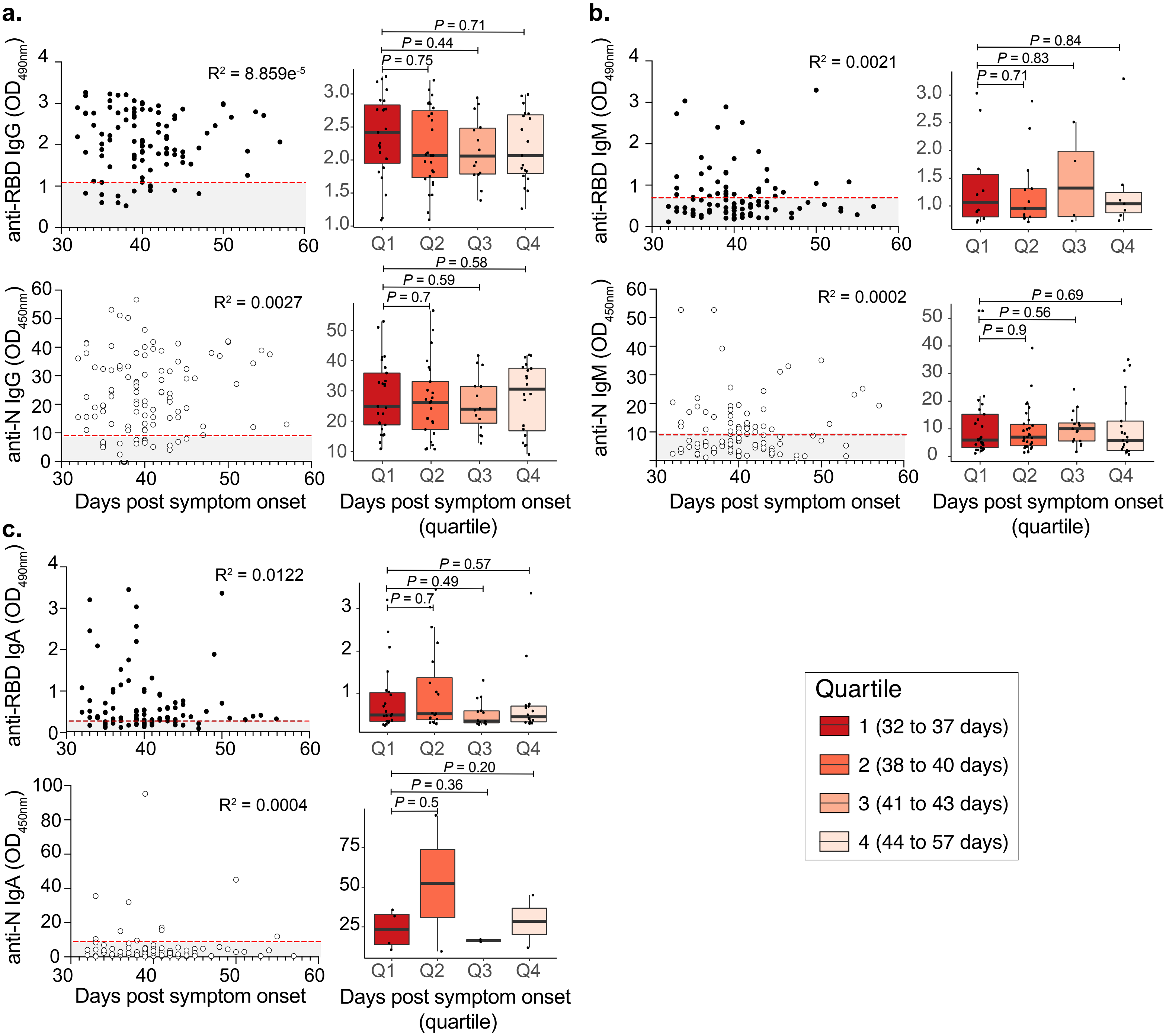


**Supplementary Figure 3. Correlations between isotype composition of SARS-CoV-2 convalescent serum and days post onset of symptoms.** Correlations of anti-RBD (top) or anti-N (bottom) IgG **(a)**, IgM **(b)** and IgA **(c)** over days post onset of symptoms are shown. Quartiles were defined based on positive samples (data points > cutoff) for anti-RBD IgG. Each quartile has the following number of observations: anti-RBD IgG (n= 29, 30, 18, 22), anti-N IgG (n= 11, 13, 5, 8), anti-RBD IgM (n= 23, 20, 15, 17), anti-N IgM (n= 11, 12, 10, 8), anti-RBD IgA (n= 24, 28, 15, 20), anti-N IgA (n= 4, 2, 2, 2). P values were calculated using the Wilcoxon rank sum test. The red dashed lines indicate the threshold (anti-N ELISA for -IgG, -IgM or -IgA are OD450nm = 9; anti-RBD ELISA for -IgG is OD490nm = 1.091; -IgA is OD490nm = 0.256 and -IgM is OD490nm = 0.694) for each ELISA.

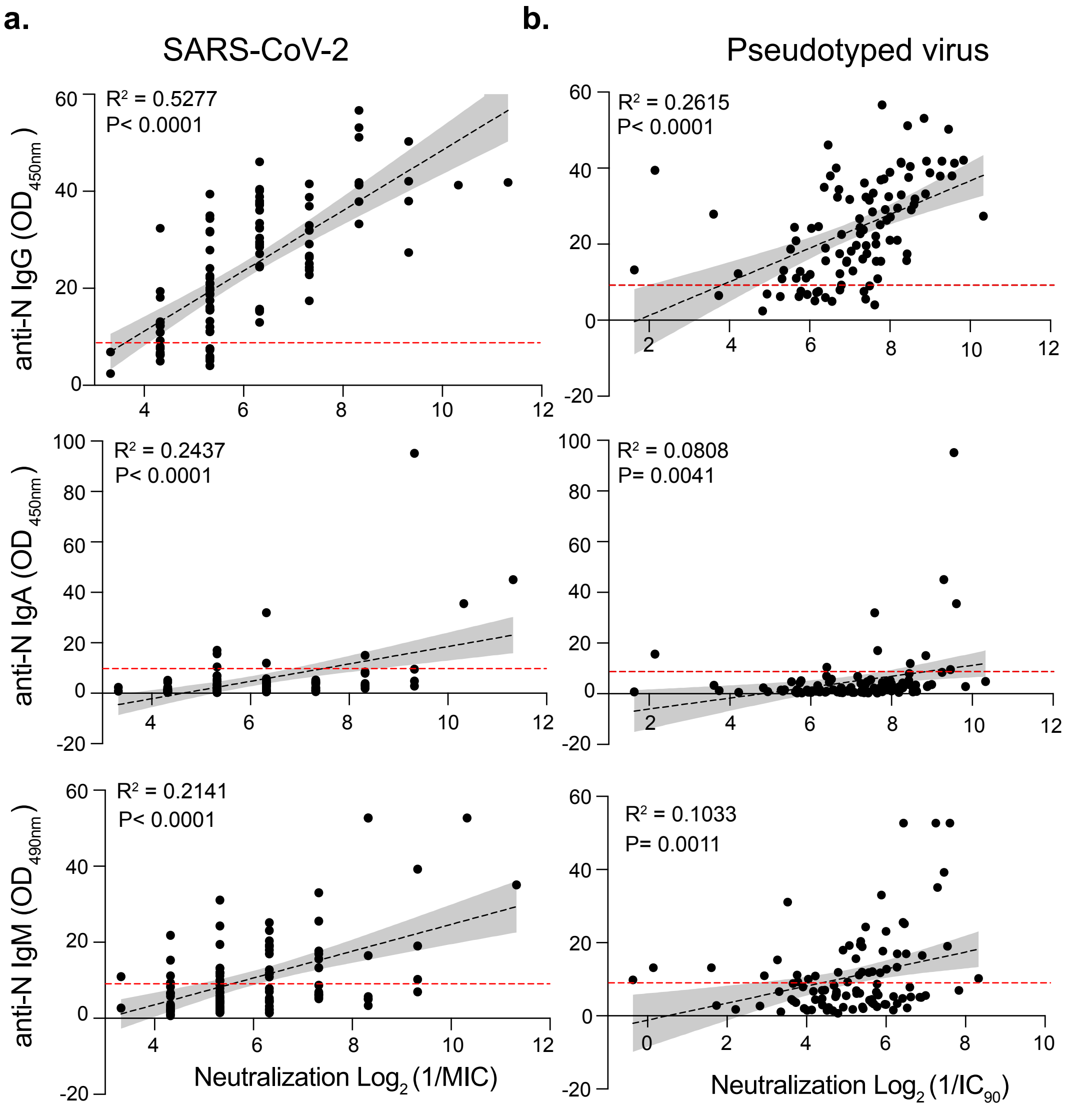


**Supplementary Figure 4. Correlation of anti-N antibody isotypes with viral neutralization.** Anti-N ELISA correlation for IgG (top), IgA (Middle) and IgM (Bottom) with viral neutralization using authentic SARS-CoV-2 **(a)** or Pseudotyped virus **(b)**. Correlation and linear regression analyses were performed using GraphPad Prism 8. P values were calculated using a two-sided F-test. The red dashed lines indicate the threshold (anti-RBD ELISA for -IgG; -IgM and -IgA is OD450nm > 9) for each ELISA.

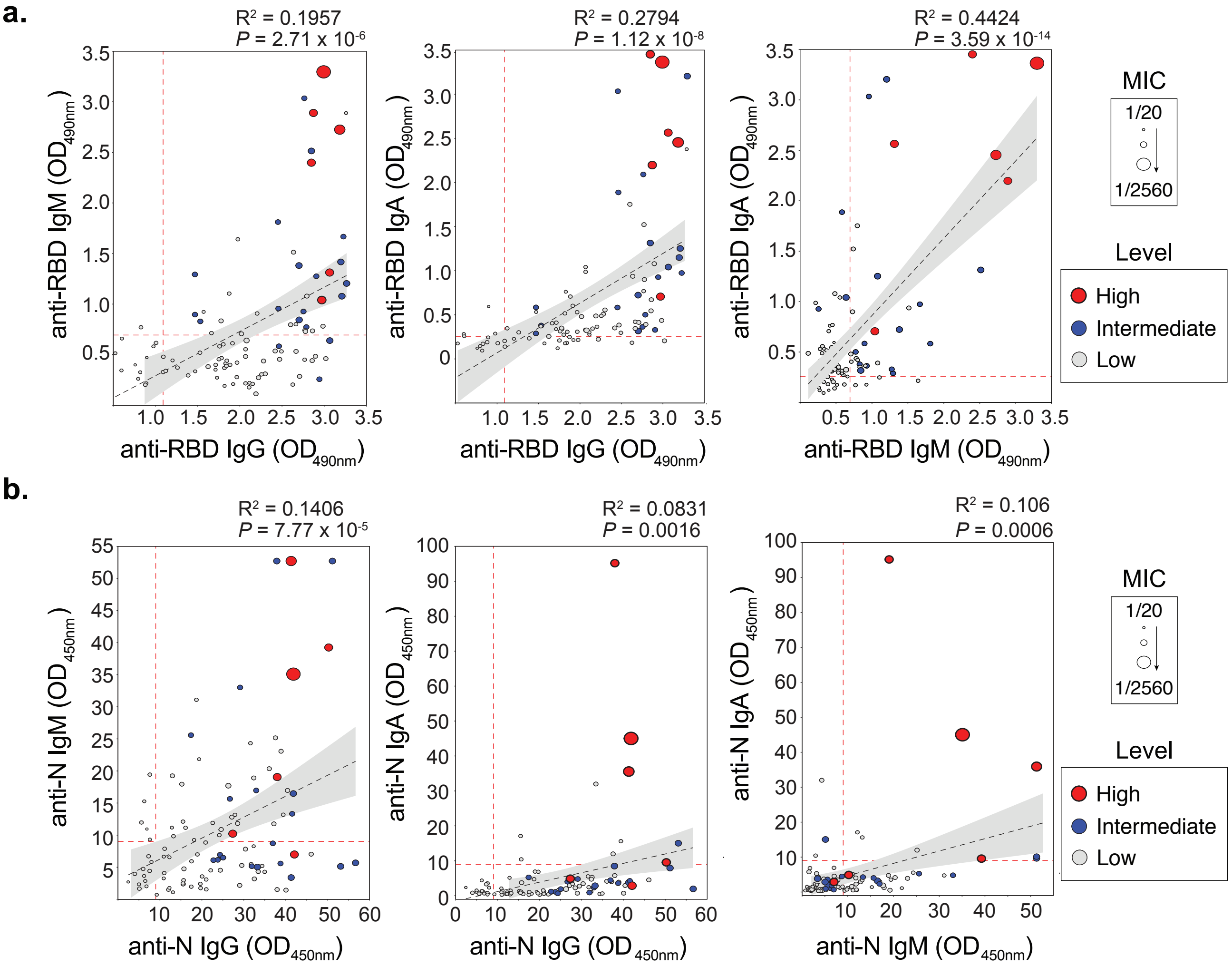


**Supplementary Figure 5. a.** Correlation analysis of anti-RBD IgM vs IgG (left panel), anti-RBD IgA vs IgG (middle panel) and anti-RBD IgA vs IgM (right panel). **b.** Correlation analysis of anti-N IgM vs IgG (left panel), anti-N IgA vs IgG (middle panel) and anti-N IgA vs IgM (right panel). For (**c**) and (**d**) correlation and linear regression analyses were performed using the linear model function in R (lm). P values were calculated using a two-sided F-test. The size of the dots indicates the MIC and the color of the dots indicates the neutralization category: High (red dots), Intermediate (blue) and Low (gray) as determined using authentic SARS-CoV-2 neutralization (see Fig. 2 legend).

**Supplementary Table 1:** SARS-CoV-2 positive NYU healthcare worker information and data used in this study.

| COVID-19 Patient # | Sympt. onset date | Days post sympt. onset | Anti-N ELISA* | | | Anti-RBD ELISA** | | | Neutralization SARS-CoV-2 | Neutralization Pseudotyped virus | |
|---|---|---|---|---|---|---|---|---|---|---|---|
| | | | IgG | IgA | IgM | IgG | IgA | IgM | 1/MIC | 1/IC90 | 1/IC50 |
| **1** | 3/16/20 | 43 | 31.59 | 5.54 | 24.32 | 2.152 | 0.344 | 0.211 | 40 | 44.9 | 108 |
| **2** | 3/18/20 | 41 | 18.14 | 0.58 | 9.26 | 0.892 | 0.244 | 0.217 | 20 | 32.83 | 125 |
| **3** | 3/17/20 | 42 | 38.76 | 3.6 | 5.58 | 2.943 | 0.927 | 0.257 | 160 | 127.7 | 361 |
| **4** | 3/12/20 | 47 | 9.24 | 0.43 | 0.71 | 1.921 | 0.214 | 0.209 | 20 | 27.7 | 61 |
| **5** | 3/20/20 | 39 | 7.56 | 0.77 | 4.42 | 0.88 | 0.19 | 0.202 | 40 | 18.69 | 44 |
| **6** | 3/19/20 | 40 | 37.18 | 4.03 | 11.76 | 2.857 | 0.545 | 0.441 | 80 | 57.75 | 121 |
| **7** | 3/15/20 | 44 | 17.47 | 0.31 | 1.97 | 2.627 | 0.183 | 0.204 | 40 | 42.9 | 121 |
| **8** | 3/19/20 | 40 | 7.58 | 3.54 | 19.43 | 2.068 | 0.987 | 0.216 | 40 | 40.93 | 95 |
| **9** | 3/8/20 | 51 | 27.15 | 2.92 | 12.8 | 2.666 | 0.345 | 0.532 | 80 | 64.58 | 181 |
| **10** | 3/18/20 | 41 | 41.83 | 3.23 | 16.48 | 2.848 | 1.314 | 2.514 | 320 | 120.4 | 1094 |
| **11** | 3/17/20 | 42 | 24.15 | 1.14 | 10 | 1.783 | 0.338 | 0.466 | 40 | 16.5 | 59 |
| **12** | 3/11/20 | 48 | 37.95 | 5.78 | 1.52 | 2.281 | 0.507 | 0.437 | 80 | 22.88 | 94 |
| **13** | 3/9/20 | 50 | 41.84 | 45.02 | 35.09 | 2.993 | 3.364 | 3.296 | 2560 | 157.1 | 1919 |
| **14** | 3/17/20 | 42 | 24.4 | 0.74 | 4.53 | 2.301 | 0.555 | 0.31 | 80 | 12.31 | 77 |
| **15** | 3/16/20 | 43 | 23.79 | 1.11 | 6.15 | 2.452 | 0.583 | 1.811 | 160 | 40.52 | 107 |
| **16** | 3/19/20 | 40 | 7.29 | 3.29 | 6.7 | 2.068 | 1.041 | 0.553 | 40 | 18.01 | 97 |
| **17** | 3/28/20 | 41 | 15.5 | 17.09 | 12.11 | 1.008 | 0.178 | 0.616 | 40 | 50.47 | 163 |
| **18** | 3/13/20 | 46 | 29.12 | 4.8 | 33.01 | 1.532 | 0.379 | 0.829 | 160 | 58.97 | 170 |
| **19** | 3/24/20 | 35 | 5.98 | 0.95 | 4.73 | 1.091 | 0.204 | 0.328 | 40 | 20.99 | 91 |
| **20** | 3/20/20 | 39 | 17.41 | 5.26 | 25.58 | 2.788 | 0.501 | 0.773 | 160 | 86.11 | 282 |
| **21** | 3/18/20 | 41 | 5.09 | 1.08 | 1.66 | 1.388 | 0.233 | 0.295 | 40 | 17.51 | 63 |
| **22** | 3/20/20 | 39 | 37.97 | 95.17 | 19.05 | 3.064 | 2.564 | 1.312 | 640 | 187.3 | 4752 |
| **23** | 3/4/20 | 55 | 37.55 | 11.99 | 25.16 | 2.708 | 0.414 | 0.285 | 80 | 88.19 | 299 |
| **24** | 3/2/20 | 57 | 12.97 | 0.52 | 19.24 | 2.068 | 0.328 | 0.482 | 80 | 33.73 | 305 |
| **25** | 3/6/20 | 53 | 34.37 | 0.52 | 5.2 | 1.846 | 0.307 | 0.44 | 40 | 26.55 | 116 |
| **26** | 3/23/20 | 36 | 13.11 | 1.51 | 1.09 | 2.114 | 0.275 | 0.222 | 20 | 10.23 | 92 |
| **27** | 3/20/20 | 39 | 7.6 | 0.34 | 9.31 | 1.712 | 0.338 | 0.351 | 20 | 13.78 | 52 |
| **28** | 3/24/20 | 35 | 17.53 | 1.08 | 5.92 | 1.884 | 0.707 | 0.399 | 40 | 26.87 | 91 |
| **29** | 3/25/20 | 34 | 32.97 | 2.34 | 16.96 | 2.764 | 2.091 | 3.035 | 160 | 77.9 | 195 |
| **30** | 3/22/20 | 37 | 32.52 | 5.29 | 3.18 | 2.772 | 1.522 | 0.74 | 80 | 62.62 | 221 |
| **31** | 3/20/20 | 39 | 6.22 | 4.4 | 3.64 | 1.471 | 0.532 | 0.335 | 20 | 13.31 | 65 |
| **32** | 3/20/20 | 39 | 26.69 | 4.03 | 15.65 | 2.457 | 3.034 | 0.956 | 160 | 37.89 | 131 |
| **33** | 3/20/20 | 39 | 21.03 | 1.2 | 3.09 | 1.752 | 0.209 | 0.455 | 40 | 63.95 | 182 |
| **34** | 3/21/20 | 38 | 50.25 | 9.56 | 39.24 | 2.848 | 3.452 | 2.398 | 640 | 175.9 | 1509 |
| **35** | 3/23/20 | 36 | 22.76 | 1.2 | 6.1 | 1.469 | 0.584 | 0.896 | 160 | 38.32 | 117 |
| **36** | 3/16/20 | 43 | 4.01 | 0.86 | 3.96 | 1.528 | 0.37 | 0.519 | 40 | 49.28 | 145 |
| **37** | 3/15/20 | 44 | 6.89 | 0.86 | 10.95 | 0.898 | 0.594 | 0.472 | Neg | 7.66 | 34 |
| **38** | 3/19/20 | 40 | 16.11 | 0.49 | 11.15 | 2.11 | 0.303 | 0.177 | 40 | 40.05 | 150 |
| **39** | 3/21/20 | 38 | 29.52 | 2.77 | 5.28 | 2.602 | 1.752 | 0.796 | 80 | 73.12 | 272 |
| **40** | 3/19/20 | 40 | 6.5 | 1.26 | 2.85 | 1.203 | 0.228 | 0.443 | 20 | 3.33 | 36 |
| **41** | 3/15/20 | 44 | 34.99 | 0.65 | 4.28 | 1.76 | 0.313 | 0.582 | 40 | 20.6 | 92 |
| **42** | 3/16/20 | 43 | 5.59 | 0.58 | 11.97 | 1.804 | 0.274 | 0.602 | 40 | 42.1 | 248 |
| **43** | 3/24/20 | 35 | 32.38 | 4.74 | 18.91 | 2.019 | 0.48 | 0.706 | 80 | 42.33 | 152 |
| **44** | 3/14/20 | 45 | 32.36 | 1.14 | 8.27 | 1.829 | 0.761 | 0.413 | 20 | 25.9 | 88 |
| **45** | 3/19/20 | 40 | 40.06 | 0.74 | 1.43 | 1.624 | 0.255 | 0.382 | 80 | 25.36 | 121 |
| **46** | 3/16/20 | 43 | 15.22 | 1.26 | 17.94 | 1.778 | 0.35 | 0.834 | 80 | 30.3 | 122 |
| **47** | 3/20/20 | 39 | 27.9 | 3.38 | 13.16 | 1.664 | 0.404 | 0.713 | 40 | 3.04 | 60 |
| **48** | 3/20/20 | 39 | 24.34 | 0.68 | 6.93 | 1.468 | 0.289 | 1.293 | 160 | 38.18 | 345 |
| **49** | 3/19/20 | 40 | 19.7 | 6.86 | 1.74 | 1.965 | 0.53 | 0.278 | 40 | 35.9 | 148 |
| **50** | 3/27/20 | 32 | 15.54 | 1.08 | 2.38 | 2.191 | 0.487 | 0.113 | 80 | 30.31 | 137 |
| **51** | 3/23/20 | 36 | 24.6 | 1.6 | 10.88 | 2.221 | 0.223 | 0.272 | 80 | 18.7 | 83 |

| | | | | | | | | | | | |
|---|---|---|---|---|---|---|---|---|---|---|---|
| **52** | 3/27/20 | 32 | 36.07 | 4.15 | 20.35 | 2.896 | 1.079 | 0.484 | 80 | 41.24 | 235 |
| **53** | 3/15/20 | 44 | 41.3 | 3.81 | 3.39 | 2.701 | 0.723 | 1.381 | 320 | 77.67 | 1101 |
| **54** | 3/20/20 | 39 | 56.66 | 1.97 | 5.68 | 2.703 | 0.317 | 0.844 | 320 | 55.85 | 765 |
| **55** | 3/19/20 | 40 | 13.23 | 0.74 | 9.83 | 1.094 | 0.139 | 0.479 | 40 | 0.776 | 10 |
| **56** | 3/19/20 | 40 | 11.12 | 1.48 | 7.88 | 1.977 | 0.215 | 1.641 | 40 | 15.02 | 53 |
| **57** | 3/18/20 | 41 | 12.85 | 5.14 | 11.17 | 0.977 | 0.287 | 0.582 | 20 | 13.61 | 38 |
| **58** | 3/26/20 | 33 | 41.54 | 4.27 | 13.32 | 2.754 | 0.363 | 0.927 | 160 | 77.31 | 243 |
| **59** | 3/19/20 | 40 | 46.08 | 4.55 | 7.03 | 2.985 | 0.436 | 0.781 | 80 | 21.98 | 524 |
| **60** | 3/14/20 | 45 | 18.7 | 4.74 | 31.09 | 1.714 | 0.411 | 0.399 | 40 | 11.49 | 48 |
| **61** | 3/20/20 | 39 | 27.36 | 4.89 | 10.22 | 2.872 | 2.197 | 2.89 | 640 | 321.3 | 785 |
| **62** | 3/23/20 | 36 | 31.91 | 0.92 | 5.15 | 3.227 | 0.974 | 1.667 | 160 | 98.68 | 1729 |
| **63** | 3/18/20 | 41 | 39.41 | 15.62 | 13.18 | 1.967 | 0.308 | 0.421 | 40 | 1.105 | 27 |
| **64** | 3/15/20 | 44 | 15.48 | 0.86 | 2.49 | 1.874 | 0.248 | 1.104 | 40 | 54.83 | 253 |
| **65** | 3/22/20 | 37 | 6.26 | 1.41 | 15.27 | 0.598 | 0.282 | 0.344 | 20 | 9.62 | 36 |
| **66** | 3/20/20 | 39 | 26.32 | 1.91 | 17.7 | 2.08 | 0.324 | 0.64 | 160 | 60.52 | 239 |
| **67** | 3/9/20 | 50 | 42.07 | 2.89 | 6.98 | 2.971 | 0.706 | 1.04 | 640 | 227.7 | 577 |
| **68** | 3/6/20 | 53 | 12.03 | 0.52 | 1.5 | 1.261 | 0.301 | 0.372 | 40 | 16.19 | 88 |
| **69** | 3/26/20 | 33 | 15.64 | 10.42 | 2.97 | 1.818 | 0.516 | 0.369 | 40 | 21.06 | 56 |
| **70** | 3/21/20 | 38 | 33.25 | 2.86 | 5.03 | 3.209 | 1.252 | 1.078 | 320 | 118.5 | 420 |
| **71** | 3/26/20 | 33 | 22.59 | 0.71 | 11.9 | 2.768 | 0.768 | 0.45 | 40 | 27.79 | 94 |
| **72** | 3/23/20 | 36 | 25.07 | 1.91 | 6.49 | 2.906 | 0.329 | 1.275 | 160 | 54.04 | 149 |
| **73** | 3/19/20 | 40 | 21.05 | 0.55 | 1.55 | 1.84 | 0.181 | 0.388 | 40 | 72.73 | 178 |
| **74** | 3/21/20 | 38 | 12.26 | 0.98 | 6.55 | 0.525 | 0.175 | 0.514 | 20 | 26.93 | 88 |
| **75** | 3/24/20 | 35 | 6.79 | 0.62 | 2.02 | 1.73 | 0.121 | 0.272 | 20 | 15.35 | 52 |
| **76** | 3/23/20 | 36 | 40.43 | 2.95 | 16.96 | nd | nd | nd | 80 | 90.81 | 324 |
| **77** | 3/16/20 | 43 | 22.11 | 0.4 | 4.51 | 2.205 | 0.364 | 0.907 | 40 | 50.05 | 158 |
| **78** | 3/22/20 | 37 | 51.14 | 7.93 | 52.71 | 3.197 | 1.149 | 1.417 | 320 | 86.66 | 716 |
| **79** | 3/26/20 | 33 | 11.02 | 0.31 | 4.09 | 0.829 | 0.171 | 0.771 | 40 | 12.67 | 60 |
| **80** | 3/20/20 | 39 | 20.06 | 0.31 | 3.56 | 2.665 | 0.421 | 0.801 | 40 | 50.24 | 171 |
| **81** | 3/22/20 | 37 | 2.43 | 2.37 | 2.71 | 0.76 | 0.121 | 0.399 | Neg | 7.09 | 35 |
| **82** | 3/24/20 | 35 | 19.35 | 1.11 | 21.83 | 0.603 | 0.257 | 0.659 | 20 | neg | neg |
| **83** | 3/26/20 | 33 | 37.87 | 8.49 | 52.71 | 3.265 | 3.204 | 1.204 | 320 | 152.8 | 2819 |
| **84** | 3/26/20 | 33 | 41.3 | 35.55 | 52.71 | 3.184 | 2.454 | 2.725 | 1280 | 195.1 | 538 |
| **85** | 3/19/20 | 40 | 10.9 | 2.12 | 3.26 | 1.819 | 0.177 | 0.191 | 20 | 51.81 | 132 |
| **86** | 3/24/20 | 38 | 10.92 | 1.01 | 6.65 | nd | nd | nd | 20 | 9.92 | 49 |
| **87** | 3/17/20 | 45 | 7.98 | 2.31 | 5.82 | 1.571 | 0.176 | 0.923 | 20 | 26.96 | 83 |
| **88** | 3/8/20 | 54 | 38.89 | 3.91 | 23.07 | 2.794 | 0.391 | 1.079 | 80 | 63.96 | 250 |
| **89** | 3/26/20 | 36 | 53.11 | 15.04 | 5.13 | 3.066 | 1.041 | 0.639 | 320 | 115.4 | 465 |
| **90** | 3/22/20 | 40 | 30.4 | 3.47 | 7.82 | 3.019 | 0.205 | 0.461 | 80 | 96.81 | 311 |
| **91** | 3/15/20 | 47 | 12.26 | 0.55 | 1.78 | 0.82 | 0.094 | 0.326 | 20 | 4.66 | 36 |
| **92** | 3/27/20 | 35 | 19.63 | 0.72 | 2.41 | 2.257 | 0.488 | 0.305 | 40 | 42.43 | 135 |
| **93** | 3/17/20 | 45 | 29.03 | 0.74 | 2.17 | 2.861 | 0.671 | 0.735 | 80 | 92.69 | 270 |
| **94** | 3/28/20 | 34 | 15.73 | 3.75 | 4.75 | 2.471 | 0.384 | 0.454 | 80 | 85.51 | 218 |
| **95** | 3/20/20 | 42 | 31.75 | 2.45 | 5.49 | 2.493 | 0.296 | 0.532 | 40 | 31.98 | 125 |
| **96** | 3/20/20 | 42 | 28.53 | 2.27 | 11.82 | 2.777 | 0.899 | 0.729 | 80 | 47.39 | 176 |
| **97** | 3/25/20 | 37 | 33.44 | 31.98 | 4.32 | 2.419 | 0.351 | 0.536 | 80 | 47.98 | 170 |
| **98** | 3/19/20 | 43 | 20.89 | 1.54 | 8.85 | 1.913 | 0.602 | 0.612 | 40 | 12.62 | 55 |
| **99** | 3/13/20 | 49 | 36.93 | 4.47 | 8.72 | 2.463 | 1.887 | 0.583 | 160 | 54.63 | 299 |
| **100** | 3/28/20 | 34 | 18.94 | 6.92 | 6.99 | 1.122 | 0.347 | 0.339 | 40 | 21 | 111 |
| **101** | 3/27/20 | 35 | 4.99 | 1.54 | 2.33 | 0.796 | 0.147 | 0.261 | 20 | 23.55 | 68 |

*Threshold for Anti-NP ELISA (IgG, IgM, IgA $OD_{450} < 9$).
**Threshold for Anti-RBD ELISA (-IgG is OD490nm = 1.091; -IgA is OD490nm = 0.256 and -IgM is OD490nm = 0.694). nd. measures not included in the analysis.
